# Rheumatism in Domestic Animals1Presented before the meeting of the American Veterinary Medical Association, New York, September, 1899.

**Published:** 1900-02

**Authors:** Herman Wellner

**Affiliations:** New York City, N. Y.


////# THE JOURNAL

OF

### COMPARATIVE MEDICINE AND VETERINARY ARCHIVES.

VOL. XXI. *FEBRUARY, 1900.* No. 2.

### RHEUMATISM IN DOMESTIC ANIMALS.[1]

BY HERMAN WELLNER, M.D., V.S.,
NEW YORK CITY, N. Y.

IT is with great reluctance that I received and accepted the invitation to address you at this time. I was first surprised that I should have the privilege of this opportunity, then I was dismayed at the thought of the undertaking; but at last, Mr. President and gentlemen, I was pleased and am pleased to-day to be accorded the honor of endeavoring to entertain you at this time. I chose the subject "Rheumatism in Domestic Animals," hoping that in the broad field which it opens to our view I might be able, out of the little experience which I had, to offer you something of note.

What does the word rheumatism signify? Does it signify the characteristic signs of the disease or the etiological name for it? Some authors give the origin for the word rheumatism from the Greek *rheo*, run, since the pain spreads from one part of the body to the other, and therefore seems to run through the body. Prof. Fröhner says that the term rheumatism should refer to muscular rheumatism only, while the term arthritis should be given to that constitutional disease which manifests itself in the fibrous tissues about the joints and sometimes in the ends of the bones. Like syphilis, it simulates many other diseases. The term rheumatism may also refer to an arthritis or to the infectious diseases—endocarditis, pleuritis, gonorrhœa rheumatica, etc.

There are several theories concerning the etiology of rheumatism, and I will try to explain one after the other. The first one is the lactic-acid theory—*i. e.*, there is a deposit of lactic acid in the

[1] Presented before the meeting of the American Veterinary Medical Association, New York, September, 1899.

affected parts of the body. This deposit is formed by the activity of the muscle through the transformation of glycogen in sugar and lactic acid. In general, the lactic acid is excreted by the body through kidney and skin (the lactic acid giving the peculiar odor to the sweat). When the muscular activity is diminished by exposure to cold the lactic acid will be retained and deposited in some parts of the body and gives rise to the pathological changes. The proofs for this theory are:

(*a*) Lactic acid was found in the urine of patients suffering from this disease.

(*b*) In diabetes mellitus where lactic acid was therapeutically given in large doses there has been observed a polyarthritis—an inflammation of more or less joints in the body.

This theory is disputed by some authors, who say:

1. It has never been proved that a larger excretion of lactic acid through the kidneys was presented by patients suffering from rheumatism.

2. Heinzeman and Senator (*Ziemssen's Encyclopædia*) say that the cause of arthritis is an increased secretion of lactic acid whereby the solid substance of the bones is dissolved, and therefore distorted and deformed joints result; this disease is different from rheumatism.

3. It is incredible that through exposure to cold and a deposit of lactic acid in the body rheumatism should exist for a long time, and sometimes for life, while lactic acid is continually excreted from the body.

4. We find cases of rheumatism in which neither exposure to cold nor strong muscular activity can be proved; therefore formation of lactic acid cannot take place.

5. Why should only lactic acid be the cause of rheumatism and not also the other chemical substances or toxic substances which are formed during the muscular activity?

The second theory is from Lotham (*British Medical Journal*, 1880), who says rheumatism will be produced through hyperoxidation of the muscles. In the normal condition there exists a nervous centre for the tissues of the muscles; if this centre is affected there will be an impediment to the chemical change. As in the normal condition, through the aid of the nervous centre, the muscles receive the oxygen through the blood circulation and give up carbon dioxide, there is in a diseased condition a retention of $CO_2$ in the tissues of the muscles; the blood will be oxidized or deoxidized, and that produces rheumatism, as there is now no activity of the nerves. But against this theory are the following objections:

1. It has not been proved that there exists such a centre which produces those chemical reactions.

2. Zunt proved that in fibroma there exists an oxidation of the tissues of the muscles, and therefore an augmented activity of the nerves, although rheumatism is not present.

A third theory is that rheumatism is a neurosis—*i. e.*, that exposure to cold affects the vasomotor nerves or the trophic nerves, whereby the centres of those nerves will be irritated, and therefore cause the inflammation. This means that there does not exist such a centre like a nervous centre which will be affected, but a trophic disturbance of different centres, like a sweat-centre, heat-centre—a condition which we find in human beings, as people affected with rheumatism perspire very heavily. In regard to this theory, rheumatism is nothing else than a neurosis catarrhalis; and as we know that when a number of persons are exposed to cold only a minority suffer from rheumatism, so we must come to the conclusion that there exists a rheumatic diathesis or disposition in different individuals who will be easier affected than others.

A fourth theory is that rheumatism is a miasmatic disease; there is a bacillus which produces this disease. Nuclagen, an English author, compares this bacillus with the bacillus of malaria. But against this theory there are the following objections:

1. We know that rheumatism is, in most part of the cases, hereditary—*i. e.*, the disposition to the disease.

2. If rheumatism were miasmatic there must be a country where this disease does not exist, like malaria; but, on the contrary, we find this disease in all countries.

3. Persons contract this disease by exposure to cold or to dampness.

A fifth theory is that muscle rheumatism and arthritis are two different diseases.

Muscle rheumatism is nothing else than an inflammation of the muscles—a myositis. The exposure to cold irritates the trophic nerves of the skin, which in a reflected way irritate the muscle nerves, whereby the vasomotor nerves also become irritated; this disease has, therefore, all the characteristics of a nutrition disorder, depending upon disturbed functions of the nerves that control nutrition.

Arthritis is a miasmatic disease; the micrococcus enters the body through the large pores of the sweat-glands, whence it is circulated through the blood-current into the heart, where they produce an endocarditis, and into the joints, through the capillary emboli, an arthritis. As a proof that arthritis is an infectious disease, Prof. Fröhner says that the following pathological conditions exist:

(*a*) General fever; (*b*) the synchronous affection of several joints, although situated some distance from one another; (*c*) existence of endocarditis; (*d*) as an arthritis, for example, has been observed in cows after confinement; the retention of the after-birth has been the cause of it.

Recklinghausen and Klebs say that the specific poison of the micrococcus of arthritis has the same effect on the joints and the fibrous structures as vaccination, as the whole body is affected at once.

Fleishhauer reports that he found in a case of arthritis acuta miliary abscesses in the heart, kidneys, and liver. A similar report of two cases I have to communicate to you:

CASE I.—A strong, well-nourished eight-year-old horse, which had been used for light work, was found one morning in the stall in such a condition that it could not be moved from one side to the other; it could not stand on its legs, but kept its position by leaning against the partition; it stood there with drooped head, which could not be lifted upward; the superficial temperature of the skin was not evenly distributed; the pulse was 85, strong, and there was marked palpitation of the heart; auscultation of the heart revealed nothing extra; but after four days there were, on auscultation of the heart, perceptible plain friction-sounds; palpitation of the heart was very strong; pulse weak; temperature 107° F.; when it was tried to move the horse it showed great pain by groaning; percussion revealed pleuritis. The treatment consisted in giving large doses of salicylic acid and fomentation of the body; the pain disappeared, but as endocarditis was present the horse died after nine days. My diagnosis of rheumatismus universalis was based on the following symptoms: (*a*) Pain all over the body; (*b*) disappearance of pain after the administration of the salicylic acid; (*c*) affection of the serous structures.

The post-mortem revealed small abscesses immediately under the pleura pulmonalis; also similar abscesses were found in the liver, spleen, and kidneys. In the left ventricle of the heart there was a fibrinous deposit upon which coagula formed; further, there were present the pathological signs of pericarditis and pleuritis.

CASE II. was a seven-year-old horse, which after three days'

illness was brought to me for treatment. Upon inquiry I found that the horse was very often shoulder-lame, dependent upon the weather. On examination I discovered pulse, 69, weak, heart beat rapid and diastolic, and post-systolic murmurs, temperature 105° F., respiration 30 and shallow. Apparently there was present an inflammation of the respiratory muscles. On the right side of the lungs friction-sounds were heard; left side was negative. Percussion caused great pain. From the nostrils there escaped liquid, which blistered the skin, also on the septum you could see superficial ulcers. In the cavity of the mouth there were also ulcers, caused by licking of the liquid which was running from the nostrils. The patient could hardly pick up his food; defecation was difficult and scanty; tenesmus was present; feces contained mucous membrane; later on the feces were mixed with blood.

In this case I also diagnosed rheumatism in connection with endocarditis, pleuritis, pneumonia infectiosa, and intestinal auto-intoxication. The post-mortem revealed abscesses in all parts of the intestines, but more developed in the large intestines; larynx, pharynx, œsophagus, and stomach were covered with ulcers; in the lungs, liver, and kidneys were miliary abscesses like those found in glanders, without their characteristics. Although this may be mistaken for acute glanders, the post-mortem proved otherwise; and I would like to impress upon the members of this association, whenever they come across such cases, to make a thorough examination, and not to condemn such patients as affected with glanders.

There is one form of rheumatic poisoning in which the rheumatic poison does seem to be present as a permanent element; it manifests itself in skin eruptions and in a tendency to repeated attacks of tonsillitis, neuritis, and anæmia.

Drs. Barlow and Warner (*Transactions of the International Medical Congress,* 1881) have drawn attention to the development of fibrous nodules in the subcutaneous tissues in connection with rheumatism. In many cases you find such fibrous and tendinous nodules under the skin, in the muscles, or tendons; so among cows along the spinal column, among horses on the shoulder, among dogs on the tendons. In children they can be seen most frequently and when the poison seems to be very virulent; the disease runs a fatal course, with chorea, pleurisy, endocarditis, pericarditis, then terminating with nephritis.

*Etiology.* (*a*) Change in the weather; damp or cold weather are frequent causes of the disease.

(*b*) Great exertion and excess of exercise also influence; for example, hunting dogs are more disposed to this disease than pet dogs.

(*c*) Age; horses in middle age are more apt to contract this disease than old or young ones.

(*d*) Diathesis; that rheumatic diathesis is of a hereditary nature; this has been certified by all authors. Some individuals are hardly ever free from rheumatic manifestations; some do not acquire this disease at all; there is always a predisposition present.

(*e*) Natural immunity; some animals having more cellular activity than others, and as a consequence a greater power to resist the invasion of the disease.

*Semiology.* Symptoms are different. In light cases there are none, but in chronic cases we find pain in almost every part of the body, tenderness on pressure over the muscles to which the inflamed nerves are distributed; but a very characteristic symptom is the change of the seat of the pain; the latter disappears after exercise or may be more easy; sometimes you can hear a peculiar crepitation in the joints; also fever is present, high pulse, and rapid respiration. As complications, may follow endocarditis, pericarditis, pleuritis, pneumonia, or bronchitis. It is very important in making the diagnosis to find out if the disease is primary or secondary; this has a great deal to do with the treatment. Sometimes we find cases where we diagnosticate congestion of the lungs or pneumonia, and after giving the ordinary medicaments we find, the next day, that there is no symptom of pneumonia present; some authors signify this as a one-day pneumonia. But why can we not suppose that this was acute rheumatism and a speedy recovery followed? The history in such cases is very important.

In regard to the pathological changes, they are sometimes not very noticeable, especially in acute cases. In chronic cases you find hyperæmia, hemorrhages, serous infiltration of the tissues, maceration, and muscular disintegration. Siedamgrotzky found in a dog suffering from rheumatism all the aponeuroses, muscles, and capillaries injected; in another case he found an interstitial chronic myositis of the mastoidei.

*Therapeutics.* In regard to treatment I must refer you to the text-books. In local affections you use stimulants like spirits of camphor, olei sinapis, ammonium caustic, with massage and Priessnitz wet-packs. In shoulder rheumatism hypodermic injections of veratrin, 0.5 to 0.1 in 0.5 g. of alcohol, increasing daily 1 g. and discontinuing it every fourth or fifth day; the latest treatment is hypodermic injection of atropin sulphate, 0.05, and morphine sulphate, 0.2, in 10 g. of water; but as in some cases colic follows you must warn the owner of the animals to watch very carefully the effects. Internally the best remedies are acid salicyl, natr. salicyl., tart. stibiat, pilocarpin, 0.4 to 0.8; as the latter has a diaphoretic effect, there is danger on exposure to cold. The diet also must be carefully regulated.

Before closing this paper I would like to mention especially muscle rheumatism in dogs; more commonly you find the abdominal muscles affected than the muscles of the neck, back, and loins. Pain is very great; the abdomen is swollen and tender; at the start there exists vomiting and anorexia, acceleration of respiration, panting, irregularity of heart-beat, high temperature, great thirst, and constipation. Duration of the disease is from seven to ten days. Sometimes the patient recovers and gets another attack, especially pet, over-fed, and stout dogs. Therapy consists first in intestinal evacuation, as oil ricini, with small doses of calomel. To relieve pain you use liquor opii sed. or hydrotherapeutic measures; internally, natr. salicylic in small doses, bismuth, etc.; as food, milk, with beef-extract; in chronic cases natr. bicarb, nux vomica, rad. gent., colombo. If paralysis is present, electricity or liniments and strychnine injections.

Now, gentlemen, pleading for conservatism in your treatment of this first effort as an author, and hoping that you will excuse the rambling and somewhat disconnected nature of this discourse, and thanking you for your kind attention, I hereby submit this paper for your consideration.

